# New insights into molecular targets for *urinary incontinence*

**DOI:** 10.4103/0253-7613.69980

**Published:** 2010-10

**Authors:** Manoj K. Poonia, Ginpreet Kaur, Meena Chintamaneni, Ilesh Changela

**Affiliations:** School of Pharmacy and Technology Management, SVKM’S NMIMS, V.L. Mehta Road, Vile Parle (W), Mumbai 400 056; 1 Department of Clinical Pharmacokinetics and Biopharmaceutics, Wockhardt Ltd., Mulund-Goregaon Link Road, Bhandup (W), Mumbai 400 078, India

**Keywords:** Detrusor muscle, incontinence, molecular targets, overactive bladder

## Abstract

*Urinary incontinence* (UI) is a disease affecting quality of life of 200 million patients worldwide. It is characterized by involuntary loss of urine. The factors involved are cystitis, detrusor hyperreflexia, spinal injury, benign prostatic hyperplasia, etc. The surge in the number of reviews on this subject indicates the amount of research devoted to this field. The prevalence is increasing at an alarming rate but unfortunately, only a few medications are currently available for this condition. There are peripheral as well as central targets including cholinergic, vanilloid, prostaglandin, kinin, calcium channel, cannabinoid, serotonin, and GABA-receptors, which act by different mechanisms to treat different types of incontinence. Drugs acting on the central nervous system (CNS) increase urinary bladder capacity, volume, or pressure threshold for micturition reflex activation while peripherally acting drugs decrease the amplitude of micturition contraction and residual volume. Anticholinergic drugs specifically M3 receptor antagonists are the first choice but have frequent side effects such as dry mouth, CNS disturbances, etc. Therefore, there is a need to understand the biochemical pathways that control urinary dysfunction to determine the potential to which they can be exploited in the treatment of this condition. This article reviews the central and peripheral molecular targets and the potential therapeutic approaches to the treatment of UI.

## Introduction

*Urinary incontinence* (UI) is an involuntary bladder contraction due to overactive bladder, which leads to loss of urine. This is a worldwide common health problem having great social impact which affects quality of life.[1] It is defined by the International Continence Society as involuntary loss of urine that is a social or hygienic problem. A population study says that 20–30% of women are affected, but only 7–12% perceive it as a problem.[2] As per the World Health Organization report 1998, there are 200 million people affected by this health problem world wide.[3] There are 53% of the homebound older persons who are incontinent, and UI is one of the 10 leading diagnoses among homebound persons.[4] Studies have indicated that as many as 50% of men report leakage due to stress UI in the first few weeks following prostate surgery after removal of the catheter.[5] In approximately 20% of men, some degree of stress UI will continue to be a significant problem 1 year post-surgery.[6] This article introduces the potential targets for treatment of UI.

## Physiology

A sequence of afferent and efferent signalling in parasympathetic, sympathetic, and somatic nerves leads to sequential storage and voiding of urine.[7] For urine storage, spinal reflexes are responsible whereas for voiding, parasympathetic stimulation is responsible. During urine storage, these reflexes mediate contraction of the outflow region through somatic (striated muscle) and sympathetic (smooth muscle) nerves.[7] During voiding, distension of bladder initiates micturition through activation of mechanoreceptors on bladder wall. The bladder receives parasympathetic innervations through pelvic nerve. Acetylcholine acts on muscarinic receptors on the detrusor muscle of bladder and stimulates them which lead to bladder contraction.[8]

In general, drugs that selectively affect the sensory arm (afferent arm) of the micturition reflex can be differentiated from those interfering with the efferent arm of the reflex through an urodynamic examination. An increase in urinary bladder capacity, volume, or pressure threshold for micturition reflex activation, without major interferences with amplitude of micturition contractions implies an inhibitory effect on urinary bladder sensory nerves. In contrast, drugs affecting the efferent arm of the micturition reflex invariably decrease the amplitude of micturition contractions and if this effect is prominent then residual volume will also be increased.[9]

## Disease

UI is characterized by involuntary loss of urine due to several factors. These factors are cystitis, detrusor hyperreflexia, spinal injury, benign prostatic hyperplasia (BPH), diabetes mellitus, obesity, parkinsonism, etc. However, despite the plethora of research and validated biological targets, effective yet safe drugs for this condition are few.

Types: Urinary incontinence (UI) is of various types such as urge incontinence, stress incontinence (SI), mixed incontinence, overflow incontinence, continuous incontinence, and reflex continence [Table 1].

**Table 1:** Symptoms and pathophysiology of urinary incontinence

*Types of urinary incontinence*	*Symptoms*	*Pathophysiology*	*References*
Stress incontinence	Involuntary leakage on effort, exertion, sneezing, and coughing	Urethral smooth muscle, external urethral sphincter, inner urethral factors, pressure transmission to bladder and urethra, pelvic floor musculature: disturbance between these factors lead to stress incontinence	10–15
Urge incontinence	Involuntary leakage accompanied by, or immediately preceded by urgency	Increased afferent activity and decreased inhibitory control in CNS and/or peripheral ganglia, increased efferent stimulation of bladder	8,13–15
Mixed incontinence	Involuntary leakage leading to urgency and also with effort, sneezing or coughing	Combination of factors causing stress and urge incontinence	8,14,15
Continuous incontinence	Involuntary leakage	Results from a fistula among the ureter, bladder or urethra and the vagina, or an ectopic ureter opening into the vagina or urethra	15
Reflex incontinence	Involuntary leakage usually not associated with the desire to micturate	Detrusor hyperreflexia and/or involuntary urethral relaxation in the absence of sensation	15
Overflow incontinence	Involuntary leakage	Overdistension of bladder, uretheral blockade, poor detrusor contractility, bladder outlet obstruction	15,16

### Urge incontinence

Bladder overactivity may be the result of several mechanisms. Both myogenic[10] and neurological[11] factors are responsible for bladder overactivity. The mechanism involved in urge incontinence may be increased afferent activity, decreased inhibitory control on CNS, and/or in peripheral ganglia, increased sensitivity to efferent stimulation in the detrusor. These mechanisms may contribute singly or in combination. Due to several mechanisms, there are many targets for treatment of UI.[12]

### Stress incontinence

SI may be associated with disturbance among factors involved in urethral sphincter competence. These factors involve urethral smooth muscle, external urethral sphincter, inner urethral factor, pressure transmission to bladder and urethra, pelvic floor musculature, hormones, connective tissue, and nerves.[13] The urethral smooth muscle and the urethral lamina propria may contribute to the intraurethral pressure, and there is pharmacological evidence that a substantial part of urethral tone is mediated through noradrenaline stimulation of α-adrenoreceptors in the urethral smooth muscle.[8] A contributing factor to SI, mainly in elderly women with lack of oestrogen, may be lack of mucosal function. The urethral smooth muscle and urethral mucosa have, therefore, been the main targets for drug treatment of SI.

### Mixed incontinence (MI)

When the factors contributing to urge and SI occur at the same time, these lead to mixed incontinence.[8]

Less common forms of UI in females include overflow, continuous, and reflex incontinence.[15] Overflow incontinence means involuntary loss of urine associated with overdistension of the bladder resulting from inefficient bladder emptying. It may occur because of poor detrusor contractility or bladder outlet obstruction or a combination of both.[15] It can also result from mechanical obstruction or due to functional disorder. In children, it may be secondary to congenital obstruction disorders (e.g., urethral valves) or to neurogenic vesical dysfunction (myelomeningocele, Hinman syndrome), whereas in adults it may often associated with BPH, a consequence of diabetes mellitus or Parkinson’s disease.[16]

Continuous incontinence results from a fistula among the ureter, bladder or urethra, and the vagina, or an ectopic ureter opening into the vagina or urethra.[15] Reflex Incontinence is due to detrusor hyperreflexia and/or involuntary urethral relaxation in the absence of sensation. It is not usually associated with the desire to micturate. This is highly suggestive of underlying neurological pathology.[15]

### Targets for treatment of UI

Peripheral targets: Peripheral targets must be the first choice for treatment of UI because of lesser side effects. These targets are as follows:

### TRPV1 receptors

The transient potential receptor vanilloid 1 (TRPV1) receptor is a nonselective cation (Ca^2+^, Na+, Mg^2+^, etc.[17]) channel that is chemically activated by capsaicin.[18] Capsaicin and resiniferatoxin (RTX) have been the first drugs used for treatment of bladder overactivity that selectively target the sensory function of urinary bladder. Although capsaicin and RTX are selective TRPV1 agonists, and both drugs increase urinary bladder capacity following intravesical instillation in patients with bladder overactivity, there are marked differences between these drugs. The intravesical instillation of capsaicin is associated with acute symptoms such as suprapubic burning and/or painful sensations often associated with uninhibited urinary bladder contractions. In patients with spinal cord lesions, capsaicin can precipitate crises of autonomic dysreflexia. In contrast, acute symptoms associated with intravesical instillation of RTX are much milder so that the clinical use of capsaicin has been almost replaced by RTX.[19] Two mechanisms underlying the vanilloid-induced contractions have been postulated. According to the first hypothesis, capsaicin or RTX directly activate capsaicin-sensitive primary sensory neurons in the subepithelial layer of the bladder which in turn release substance P. Substance P sensitizes smooth muscle cells resulting in increased contractions.[20] The second hypothesis is based on the recent finding that TRPV1 is also expressed by epithelial cells of the transitional epithelium and activation of these TRPV1-expressing cells results in ATP release, activating P2X3 receptors expressed by bladder afferents.[21,22] Thus, RTX and its derivatives are preferred in treating UI due to milder adverse symptoms compared to capsaicin.

## Prostaglandin receptors

Distension of bladder stimulates the production of PGE2 by urothelium. PGE2 can sensitize suburothelial nerves and contribute to overactive bladder, as indicated in animal models in which PGE2 antagonists reduce bladder hyperactivity.[23] A variety of experimental bladder conditions are associated with upregulation of inflammatory mediators including PGs, leukotrienes, and a variety of cytokines.[24] Blockade of various PGs could be a useful therapeutic strategy.[25] Bladder PGs have modulatory roles in the afferent arm of the micturition reflex.[26,27] Myelinated Aδ afferent fibers are involved in the physiological triggering of the micturition reflex. Proposed etiologies for overactive bladder include altered excitability of afferent nerves thus targeting these hyperactive pathways via the PG cascade represents an attractive therapeutic approach.[28]

### Opioid receptors

Nociceptin, a OP4 receptor agonist, modulates the micturition reflex by inhibiting the activity of TRPV1-expressing neurons at the periphery.[29] A study states that the intravesical infusion of nociceptin increases the urinary bladder capacity in patients with bladder overactivity, but not in normal subjects.[30] Thus, nociceptin and its derivatives may be clinically useful in UI.

### Estrogen receptors (ER)

The ovarian cycles affect the micturition habits. Electrophysiological evidence indicates that during proestrus mechanoreceptor excitability increases and detrusor compliance decreases.[31] A recent study states that mice-lacking ER-α do not show bladder overactivity following acute intravesical infusion of capsaicin, though they show no major changes in detrusor contractility, unlike mice lacking ER-β. This suggests that ER-α are essential for the activity of TRPV1 receptors.[32] Thus drugs acting on ER-α can be used to treat UI.

### Tachykinin receptors

All tachykinin receptors (NK1, NK2, and NK3) are expressed in the urinary bladder. These receptors are potential targets for tachykinins released from a subset of TRPV1-expressing neurons. Apart from detrusor muscle (mainly NK2) and blood vessels (NK1), tachykinin receptors are also located on structures which can modulate the sensory function. There is experimental evidence indicating that peripheral stimulation of NK1, NK2, or NK3 receptors induce bladder overactivity so drugs targeted to block these receptors can be beneficial in overactive bladder.[33] Substance P is widely distributed in the body of mammals and has a variety of physiological functions including actions as a neurotransmitter- or neuromodulatormediated by the tachykinin NK 1 receptor.[34] Studies show that tachykinins- or capsaicin-sensitive neurons play a role in the micturition reflex in both the peripheral as well as central nervous system.[35,36] As detrusor muscle contains mainly NK2 receptors, specific antagonists of these receptors may be considered potential drugs for UI.

### Bradykinin receptors

Both bradykinin receptors (B1 and B2) are expressed on inflamed urinary bladder while B2 is present constitutively on the same. B2 receptor agonist, bradykinin, stimulates the micturition reflex in normal rats, prevented by capsaicin pretreatment and partly reduced by COX inhibitors. It has been recently demonstrated that bradykinin induces release of ATP from urothelial cells through the stimulation of B1 and B2 receptors,[37] suggesting that the kinin-induced component of bladder overactivity is mediated by ATP.[38] Specific antagonists of B1 receptors may be better for treatment of UI as they avoid B2-associated side effects.

### EP prostanoid receptors

COX inhibitors are known to improve bladder overactivity.[32] COX inhibitors act by blocking COX2 activity and consequently reducing the stimulation of capsaicin-sensitive fibers.[38] Recent studies show that aspirin reduces bladder overactivity in a rat model, but its effect was not additive to capsaicin pretreatment which may indicate that both drugs act on TRPV1-expressing neurons.[39] However, COX inhibitors (including selective COX-2) are not indicated for hemorrhagic cystitis because of the tissue protecting effects of prostanoid or when the urothelium is not protected, in case of interstitial cystitis. Therefore, it is possible that targeting EP receptors rather than COX would produce beneficial effects on bladder overactivity without damaging bladder tissue.[32]

### Calcium (Ca^2+^) channel

The detrusor can relax independently by the reduction in cytosolic Ca^2+^ through a mechanism termed Ca^2+^ sensitization. Myosin phosphatase regulates smooth muscle tone by shifting contractile protein kinetics.[40] A rise in cytosolic calcium increases the action potential in smooth muscle cells, which in turn is derived from entry of calcium via voltage-gated Ca^2+^ channels. Thus, blockade of Ca^2+^ channels might relax the detrusor and improve urge continence.[41] A study indicates that the inhibition of Rho kinase attenuates *in vitro* bladder contraction in tissues from healthy and diseased animals.[42]

### Potassium (K^+^) channels

The opening of K^+^ channel favors the extracellular efflux of potassium and regulates the resting potential, duration of action potentials and duration of hyperpolarisation that follows action potential.[43] NS-8 (sub type of K^+^-channel) increases urinary bladder capacity without affecting the amplitude of micturition contraction.[44] It was speculated that the main targets of NS-8 are large conductance calcium-activated K^+^ channels.[45] The opening of K^+^ channels relaxes the detrusor *in vitro*. However, the usefulness of K^+^-channel openers targeting smooth muscle is limited by hypotension and other cardiovascular side effects.[46]

### Sodium (Na^+^) channels

The two types of voltage-activated TTX-resistant Na^+^ channels Nav1.8 and Nav1.9 are expressed in urinary bladder sensory neurons.[47] Intrathecal injection of an antisense oligonucleotide blocks the expression of Nav1.8, increases the urinary bladder capacity in normal animals, and decreases bladder overactivity elicited by the intravesical instillation of diluted acetic acid in a rat model.[48]

### Cholinergic receptors

Muscarinic receptor antagonists (M3 & M2, and more recently M3-selective) are the mainstay for treatment of bladder overactivity. Antimuscarinic agents increase urinary bladder capacity. Their clinical efficacy is limited by impairment of urinary bladder contractility leading to incomplete voiding. However, unlike the increase of residual volume, the increase of urinary bladder capacity is not explained by reduction of parasympathetic efferent drive. Recently, it has been shown that intravesical oxybutynin decreases the firing of urinary bladder afferent C-fibers[49] which could be related to muscarinic receptor blockade as it selectively antagonizes the increase in frequency of micturition induced by carbachol.[50]

### Purinergic and pyrimidinergic receptors

P2X receptors present on detrusor muscle are activated by ATP.[51] P2X receptors are also present in the bladder urothelium.[52] Muscarinic receptors mediate 15% of rat urinary bladder neurogenic contraction, and another 50% is mediated by P2X receptor mechanisms.[53] Mice lacking the P2X3 receptor exhibit reduced inflammatory pain and marked urinary bladder hyporeflexia with reduced voiding frequency and increased voiding volume, suggesting that P2X3 receptors are involved in mechanosensory transduction underlying both inflammatory pain and physiological reflexes.[54]

### GABA-receptor

GABA-B receptor agonists are beneficial in the treatment of overactive bladder.[55] Baclofen, a GABA-B receptor subtype agonist, inhibits reflex activation of motor neurons.[56] Baclofen does not affect the response to stimulation of electrical field or muscarinic receptors of human detrusor strips[57] but some reduction in the nerve-mediated response occurred, with baclofen acting via. GABA-B receptors in detrusor.[58,59] This suggests a peripheral site of action for baclofen in the treatment of detrusor instability [Table 2].

**Table 2:** Drugs for treatment of UI along their molecular targets and mechanism of action

*Targets*	*Drugs*	*Mechanism*	*References*
TRPV1 receptors	Capsaicin, resiniferatoxin	Counter irritant	19
Prostaglandin receptors	Indomethacin Flurbiprofen	Blockade of PGs	25
Opoid receptors	Nociceptin (brain extract)	OP4 receptors agonist	29
Estrogen receptors (ER)	Not available	ER-α agonist	32
Tachykinin receptors	Not available	Blockade of NK1, NK2, and NK3	33
Bradykinin receptors	Not available	Blockade of B1 receptor	37,38
EP prostanoid receptors	Not available	EP prostanoid antagionsm	32
Calcium (Ca^2^+) channel	Nifedipine, verapamil, and imipramine	Blockade of Ca^2^+ channels	41,56
Potassium (K+) channels	Not available	K+-channel openers	46
Sodium (Na+) channels	Antisense oligonucleotide	Blocks the expression of Nav1.8	48
Cholinergic receptors	Oxybutynin, tolterodine, propantheline, terodiline, flavoxate, darifenacin, imipramine	Muscarinic receptor antagonistic	56
Purinergic and pyrimidinergic receptors	Not available	Afferent blockade	21,22
GABA-receptor	Baclofen	GABA-B receptor subtype agonist	56
Dopaminergic receptors	Not available	Blockade of dopamine D2 receptor	25
Cannabinoid (CB) receptors	Cannabis extract	CB1 and CB2 receptor antagonists	38,62
Serotonin receptors	Fluoxetine	5-HT reuptake blocker	67
β-adrenergic receptor	Imipramine, terbutaline, clenbuterol, and salbutamol	Adrenergic (α-2) receptor antagonistic	56

## Central Targets

Serotonin and dopamine receptor are the central targets for UI. Other available targets are described in the following sections.

### Dopaminergic receptors

Neurologic diseases lead to overactive bladder and urge incontinence by altering neurotransmission in the CNS. In Parkinson’s disease dopamine (D2) receptors in the basal ganglia and substantia nigra facilitate detrusor activity, whereas the D1 receptor facilitates micturition.[60] When drug administered to Parkinson’s patients, dopaminergic drug improves bladder activity but might worsen neurogenic detrusor overactivity and urge incontinence. Thus, selective dopamine antagonists could be useful in the treatment of urge incontinence in Parkinson’s patients. In the middle cerebral artery ligation model of stroke in rats, increased glutaminergic transmission with decreased D1 receptor activation and increased D2 receptor activity are linked with increased bladder activity.[61] Therefore, blockade of dopamine D2 receptors is a logical treatment for urge incontinence.[25]

### Cannabinoid (CB) receptors

A recent study indicated that cannabis extract, containing δ-9-tetrahydrocannibinol, reduced bladder overactivity in patients of multiple sclerosis.[62] Obviously this effect might be exerted at the CNS level, where CB1 receptors are densely expressed.[38] Pharmacological effect of cannabinoids is complicated as both CB1 and CB2 receptor antagonists possess inverse agonist activity so that the interpretation of experiments with agonists is difficult. Some cannabinoid ligands affect TRPV1-expressing neurons through an unknown mechanism (not mediated by CB1, CB2, or TRPV1 receptors). Therefore, although functional evidence indicates that cannabinoids inhibit bladder overactivity, the precise mechanism and the site of action remain undefined.

### Serotonin receptors

Aberrations in central neurotransmission have been postulated for some forms of depression and anxiety. This might influence voiding and explain the association of disparate conditions with overactive bladder and urge incontinence. For example, reduced serotonin function[63] is responsible for a subpopulation of depressed patients.[64] Because activation of subtypes of 5-HT receptors in the brain and spinal cord inhibits bladder activity,[65] it may be postulated that a reduction in the 5-HT transporter or 5-HT synthesis in the brain leads to bladder overactivity. This is supported by clinical observations that tricyclic antidepressants and the combined 5-HT/NE reuptake blockers are useful in both depression and urge incontinence. Interestingly, 5-HT function is also decreased in Alzheimer’s disease and late-life depression, both of which are associated with overactive bladder and urge incontinence.[66] Treatment that lowers 5-HT in the brain of rodents produce urinary frequency and intermicturition contractions similar to overactive bladder. This can be reversed with the 5-HT reuptake blocker fluoxetine.[67] These findings support that altered 5-HT function leads to overactive bladder and urge incontinence.[25]

### β-Adrenergic receptor

The detrusor muscle responds to adrenergic stimulation by relaxation. Bladder relaxation caused by increased hypogastric nerve activity and mediated via β-adrenergic receptor activation is important during the collecting phase of bladder filling.[68] Bladder capacity significantly increases with no change of micturition pressure or threshold pressure.[69] It was hypothesized that drug inhibits bladder functions by acting directly on the detrusor muscle. This observation was also supported by the β3-receptor over expression in the detrusor muscle.[70] β3-adrenergic receptor expressed on the urothelium may also contribute to the regulation of bladder function.[71]

## Conclusion

During the last two decades, significant clinical and scientific data on the UI has been generated. Unfortunately, the accumulated knowledge has not been translated into successful treatment of patients with functional lower UI. As evident in this review, role of the detrusor smooth muscle and the changes in diseased or abnormal states are well understood. Theoretically, this should provide targets for treatment. Anticholinergic drugs are commonly available and widely used in the treatment of UI in spite of having frequent adverse effects such as blurred vision, dry mouth, etc. Newer targets for treatment will reduce the number and frequency of adverse effects. The most important target may be specific cholinergic receptors. The drug specific to M3 receptors with antispasmodic activity may be a better treatment for UI. PG receptor may be another useful target as specific PGE2 antagonists reduce bladder hyperactivity. Regarding central targets, selective D2 antagonists may be useful in treating urge UI, especially in patients having diseases like parkinsonism.

Apart from the molecular targets mentioned earlier, some of the clinical aspects for assessment of UI may be complicated by patient’s hesitation to talk about this embarrassing condition. Gender differences between patients and physician may worsen this problem because patients may underreport symptoms of incontinence.[72] Therefore, improved physician–patient communication may help in defining the better scope of OAB (Overactive bladder) symptoms and lead to more appropriate and successful treatments.

## References

[1] Schefchyk SJ (1989). The effect of lumbosacral deafferentation on pontine micturition centre-evoked voiding in the decerebrate cat. Neurosci Lett.

[2] Quartara L, Maggi CA (1998). The tachykinin NK1 receptor: Part II. Distribution and pathophysiological roles. Neuropeptides.

[3] Steers WD (2002). Pathophysiology of overactive bladder and urge *Urinary incontinence*. Rev Urol.

[4] Martin CM (1997). *Urinary incontinence* in the elderly. Consult Pharm.

[5] Catalona WJ, Ramos CG, Carvalhal GF (1999). Contemporary results of anatomic radical prostatectomy. Ca Cancer J Clin.

[6] Burgio K, Goode P, Urban D, Umlauf M, Locher J, Bueshen A (2005). Preoperoative bio-feedback-assisted behavioral training to reduce post-prostatectomy incontinence. Birmingham VA Medical Center. Neurourol Urodyn.

[7] De Groat WC, Downie JW, Levin RM, Long Lin AT, Morrison JFB, Nishizawa O, Abrams P, Khoury S, Wein A (1999). Basic neurophysiology and neuropharmacology. Incontinence. 1^st^ International Consultation on Incontinence.

[8] Andersson KE (1993). Pharmacology of lower urinary tract smooth muscles and penile erectile tissues. Pharmacol Rev.

[9] Schefchyk SJ (1989). The effect of lumbosacral deafferentation on pontine micturition centre-evoked voiding in the decerebrate cat. Neurosci Lett.

[10] Brading AF (1997). A myogenic basis for the overactive bladder. Urology.

[11] De Groat WC (1997). A neurological basis for the overactive bladder. Urology.

[12] Andersson KE (1997). The overactive bladder: Pharmacological basis of drug treatments. Urology.

[13] De Lancey OL, Fowler CJ, Keane D, Macarak E, Mostwin J, Elbadawi A (1999). Pathophysiology. Incontinence. 1^st^ International Consultation onIncontinence.

[14] Bates P, Bradley WE, Glen E, Griffiths D, Melchior H, Rowan D (1979). The standardization of terminology of lower urinary tract function. J Urol.

[15] Brenda K, Jhuma B, Simon J (2005). Types of incontinence and clinical assessment Women�s Health Medicine.

[16] Andersson KE (2000). Drug Therapy of Urinary Urge Incontinence: A Systematic Review. Clin Obstet Gynaecol.

[17] Pedersen SF, Owsianik G, Nilius B (2005). TRP channels: An overview. Cell Calcium.

[18] Starowicz K, Nigam S, Di Marzo V (2007). Biochemistry and pharmacology of endovanilloids. Pharmacol Therap.

[19] Birder LA, Nakamura Y, Kiss S, Nealen ML, Barrick S, Kanai AJ (2002). Altered urinary bladder function in mice lacking the vanilloid receptor TRPV1. Nat Neurosci.

[20] Quartara L, Maggi CA (1998). The tachykinin NK1 receptor: Part II. Distribution and pathophysiological roles. Neuropeptides.

[21] Birder LA, Kanai AJ, De Groat WC, Kiss S, Nealen ML, Burke NE (2001). Vanilloid receptor expression suggests a sensory role for urinary bladder epithelial cells. Proc Natl Acad Sci USA.

[22] Ferguson DR, Kennedy I, Burton TJ (1997). ATP is released from rabbit urinary bladder epithelial cells by hydrostatic pressure changes-a possible sensory mechanism. J Physiol.

[23] Apodaca G (2004). The uroepithelium: Not just a passive barrier. Traffic.

[24] Azadzoi KM, Shinde VM, Tarcan T, Kozlowski R, Siroky MB (2003). Increased leukotriene and prostaglandin release and over activity in the chronically ischemic bladder. J Urol.

[25] William DS (2004). Management of urinary incontinence in women. Drug Discov Today Ther Strateg.

[26] Mikhailidis DP, Jeremy JY, Dandona P (1987). Urinary bladder prostanoids - their synthesis, function and possible role in the pathogenesis and treatment of disease. J Urol.

[27] Maggi CA (1992). Prostanoids as local modulators of reflex micturition. Pharm Res.

[28] Steers WD (2002). Pathophysiology of overactive bladder and urge *Urinary incontinence*. Rev Urol.

[29] Lecci A, Giuliani S, Meini S, Maggi CA (2000). Nociceptin and the micturition reflex. Peptides.

[30] Lazzeri M, Calò G, Spinelli M, Guerrini R, Beneforti P, Sandri S (2001). Urodynamic and clinical evidence of acute inhibitory effects of intravesical nociceptin/orphanin FQ on detrusor hyperactivity in humans: A pilot study. J Urol.

[31] Shea VK, Cai R, Crepps B, Mason JL, Perl ER (2000). Sensory fibers of the pelvic nerve innervating the rat’s urinary bladder. J Neurophysiol.

[32] Andersson KE, Wein AJ (2004). Pharmacology of the lower urinary tract: Basis for current and future treatments of *Urinary incontinence*. Pharmacol Rev.

[33] Candenas L, Lecci A, Pinto FM, Patak E, Maggi CA, Pennefather JN (2005). Tachykinins and tachykinin receptors: Effects in the genitourinary tract. Life Sci.

[34] Otsuka M, Yoshioka K (1993). Neurotrasmitter functions of mammalian tachykinins. Physiol Rev.

[35] Maggi CA (1991). The role of peptides in the regulation of the micturition reflex: An update. Gen Pharmacol.

[36] Maggi CA (1997). Tachykinins as peripheral modulators of primary afferent nerves and visceral sensitivity. Pharmacol Res.

[37] Chopra B, Barrick SR, Meyers S, Beckel JM, Zeidel ML, Ford AP (2005). Expression and function of bradykinin B1 and B2 receptors in normal and inflamed rat urinary bladder urothelium. J Physiol.

[38] Alessandro L, Carlo AM (2005). Overactive Urinary Bladder; Targeting sensory pathways. Drug Discov Today Ther Strateg.

[39] Tsukimi Y, Mizuyachi K, Matsumoto H, Sato M, Ng B, Tajimi M (2004). Mechanism of action by which aspirin alleviates detrusor hyperactivity in rats. J Pharmacol Sci.

[40] Wibberley A, Chen Z, Hu E, Hieble JP, Westfall TD (2003). Expression and functional role of Rho-kinase in rat urinary bladder smooth muscle. Br J Pharmacol.

[41] Andersson KE, Chapple C, Wein A (2001). The basis for drug treatment of the overactive bladder. World J Urol.

[42] Stephan LM, Peters, Martina S, Martin CM (2006). Rho Kinase:a target for treating urinary bladder dysfunction?. Trends Pharmacol Sci.

[43] Alexander SP, Mathie A, Peters JA (2004). Guide to receptors and channels. Br J Pharmacol.

[44] Gopalakrishnan M, Shieh CC (2004). Potassium channel subtypes as molecular targets for overactive bladder and other urological disorders. Expert Opin Ther Targets.

[45] Meredith AL, Thorneloe KS, Werner ME, Nelson MT, Aldrich RW (2004). Overactive bladder and incontinence in the absence of the BK large conductance Ca2+- activated K+ channel. J Biol Chem.

[46] Lynch JJ, Brune ME, Lubbers NL, Coghlan MJ, Cox BF, Polakowski JS (2003). K-ATP opener-mediated attenuation of spontaneous bladder contractions in ligature-intact, partial bladder outlet obstructed rats. Life Sci.

[47] Laird JM, Souslova V, Wood JN, Cervero F (2002). Deficits in visceral pain and referred hyperalgesia in Nav1.8 (SNS/PN3)-null mice. J Neurosci.

[48] Yoshimura N, Seki S, Novakovic SD, Tzoumaka E, Erickson VL, Erickson KA (2001). The involvement of the tetrodotoxin-resistant sodium channel Na(v)1.8 (PN3/ SNS) in a rat model of visceral pain. J Neurosci.

[49] De Wachter S, Wyndaele JJ (2003). Intravesical oxybutynin: A local anesthetic effect on bladder C afferents. J Urol.

[50] Kim Y, Yoshimura N, Masuda H, de Miguel F, Chancellor MB (2005). Antimuscarinic agents exhibit local inhibitory effects on muscarinic receptors in bladder-afferent pathways. Urology.

[51] Chancellor MB, Kaplan SA, Blaivas JG (1992). The cholinergic and purinergic components of detrusor contractility in a whole rabbit bladder model. J Urol.

[52] Yoshimura N, de Groat WC (1997). Neural control of the lower urinary tract. Int J Urol.

[53] Hashimoto M, Kokubun S (1995). Contribution of P2-purinoceptors to neurogenic contraction of rat urinary bladder smooth muscle. Br J Pharmacol.

[54] Cockayne DA, Hamilton SG, Zhu QM, Dunn PM, Zhong Y, Novakovic S (2000). Urinary bladder hyporeflexia and reduced pain-related behaviour in P2X3-deficient mice. Nature.

[55] Sanger GJ, Munonyara ML, Dass N, Prosser H, Pangalos MN, Parsons ME (2002). GABA(B) receptor function in the ileum and urinary bladder of wildtype and GABA(B1) subunit null mice. Auton Autacoid Pharmacol.

[56] Rang HP, Dale MM (1991). Pharmacology.

[57] Ayyat FM, Lloyd LK, Kuhlemeier KV (1984). Effect of skeletal muscle relaxants on bladder smooth muscle. J Urol.

[58] Chen TF, Doyle PT, Ferguson DR (1992). Inhibitory role of gamma-amino-butyric acid in the rabbit urinary bladder. Br J Urol.

[59] Chen TF, Doyle PT, Ferguson DR (1994). Inhibition in the human urinary bladder by gamma-amino-butyric acid. Br J Urol.

[60] Yoshimura N, Kuno S, Chancellor MB, De Groat WC, Seki S (2003). Dopaminergic mechanisms underlying bladder hyperactivity in rats with a unilateral 6-hydroxydopamine (6-OHDA) lesion of the nigrostriatal pathway. Br J Pharmacol.

[61] Yokoyama O, Yoshiyama M, Namiki M, de Groat WC (2002). Changes in dopaminergic and glutamatergic excitatory mechanisms of micturition reflex after middle cerebral artery occlusion in conscious rats. Exp Neurol.

[62] Brady CM, DasGupta R, Dalton C, Wiseman OJ, Berkley KJ, Fowler CJ (2004). An openlabel pilot study of cannabis-based extracts for bladder dysfunction in advanced multiple sclerosis. Mult Scler.

[63] Griffiths D (1998). Clinical studies of cerebral and urinary tract function in elderly people with *Urinary incontinence*. Behav Brain Res.

[64] Arango V, Underwood MD, Mann JJ (2002). Serotonin brain circuits involved in major depression and suicide. Prog Brain Res.

[65] De Groat WC (2002). Influence of central serotonergic mechanisms on lower urinary tract function. Urology.

[66] Steers WD, Lee KS (2001). Depression and incontinence. World J Urol.

[67] Lee KS, Na YG, Dean-McKinney T, Klausner AP, Tuttle JB, Steers WD (2003). Alterations in voiding frequency and cystometry in the clomipramine induced model of endogenous depression and reversal with fluoxetine. J Urol.

[68] Maria GU, Valentina V, Emanuel R, Francesca C, Fabrizio DP (2009). Pharmacol Res.

[69] Fujimura T, Tamura K, Tsutsumi T, Yamamoto T, Nakamura K, Koibuchi Y (1999). Expression and possible functional role of the beta3-adrenoceptor in human and rat detrusor muscle. J Urol.

[70] Leon LA, Hoffman BE, Gardner SD, Laping NJ, Evans C, Lashinger ES (2008). Effects of the beta3-adrenergic receptor agonist CL-316243 on bladder micturition reflex in spontaneously hypertensive rats. J Pharmacol Exp Ther.

[71] Otsuka A, Shinbo H, Matsumoto R, Kurita Y, Ozono S (2008). Expression and functional role of beta-adrenoceptors in the human urinary bladder urothelium. Naunyn Schmiedebergs Arch Pharmacol.

[72] Diokno A, Lee P, Zorn BH, Lenderking WR, Grossman MA, Bull SA (2001). Factors associated with clinical assessment of overactive bladder and selection of treatment. Clin Ther.

